# Black Eagle

**Published:** 1848-11

**Authors:** 


*Black Eagle.*—A carved, black, two-headed eagle, on which the Austrian coat of arms is escutcheoned, has lately been displayed at the window of a store in the "Commercial buildings," in which our Journal is printed. By a card placed over the model, the admiring spectator is informed that it is the insignia of the agency for the Grafenberg patent medicines. As we looked at it, placed there for such an object, we could not but be struck with an appropriateness of the design in some respects. A double-headed monster, of the Falcon, or prey seizing genus, and black as Erebus, it is a fitting emblem of the business it denotes, while, at the same time, it is a grievous insult to the noble bird whose deformed image is prostituted to such base use. We trust that those who call to subscribe for the Buffalo Medical Journal, pay subscriptions, etc., will not suppose that our periodical is in any way implicated in the Grafenberg system.

